# Standardized Digital Colposcopy with Dynamic Spectral Imaging for Conservative Patient Management

**DOI:** 10.1155/2017/5269279

**Published:** 2017-12-25

**Authors:** Angelika Kaufmann, Christina Founta, Emmanouil Papagiannakis, Raj Naik, Ann Fisher

**Affiliations:** ^1^Northern Gynaecological Oncology Centre, Gateshead NHS Foundation Trust, Queen Elizabeth Hospital, Sheriff Hill, Gateshead NE9 6SX, UK; ^2^Musgrove Park Hospital, Taunton & Somerset NHS Foundation Trust, Taunton TA1 5DA, UK; ^3^DYSIS Medical Ltd, Edinburgh, UK

## Abstract

**Background:**

Colposcopy is subjective and management of young patients with high-grade disease is challenging, as treatments may impair subsequent pregnancies and adversely affect obstetric outcomes. Conservative management of selected patients is becoming more popular amongst clinicians; however it requires accurate assessment and documentation. Novel adjunctive technologies for colposcopy could improve patient care and help individualize management decisions by introducing standardization, increasing sensitivity, and improving documentation.

**Case:**

A nulliparous 27-year-old woman planning pregnancy underwent colposcopy following high-grade cytology. The colposcopic impression was of low-grade changes, whilst the Dynamic Spectral Imaging (DSI) map of the cervix suggested potential high-grade. A DSI-directed biopsy confirmed CIN2. At follow-up, both colposcopy and DSI were suggestive of low-grade disease only, and image comparison confirmed the absence of previously present acetowhite epithelium areas. Histology of the transformation zone following excisional treatment, as per patient's choice, showed no high-grade changes.

**Conclusion:**

Digital colposcopy with DSI mapping helps standardize colposcopic examinations, increase diagnostic accuracy, and monitor cervical changes over time, improving patient care. When used for longitudinal tracking of disease and when it confirms a negative colposcopy, it can help towards avoiding overtreatment and hence decrease morbidity related to cervical excision.

## 1. Introduction

In cytology-based cervical screening programs, women with high-grade cytology are directly referred for colposcopic examination [1, 2], to identify and treat precancerous changes of the cervix (cervical intraepithelial neoplasia, CIN) and prevent risk for progression to invasive cancer. High-grade CIN lesions identified colposcopically and/or histologically are conventionally excised. Furthermore, immediate treatment of women with high-grade cytology, even in the absence of high-grade colposcopic findings, is not unusual, as up to 84% of cases have been reported to have CIN2 or CIN3 on histology [3, 4].

However, excision of cervical tissue can result in cervical deficiency and increase the risk of miscarriage, preterm labour, preterm premature rupture of membranes [5, 6], and cervical stenosis. Furthermore, when the excision of dysplastic cells is incomplete, repeated treatment may be needed, increasing the risks. Considering that a large cohort of women undergo colposcopy during their childbearing years and that, in younger women, CIN2 has an up to 50% likelihood of regression [7–9], conservative management of CIN2 lesions is becoming increasingly popular amongst clinicians for selected patients [10].

In addition, sensitivity of colposcopy is known to be as low as 55–65% [11–13], punch biopsy has been reported to often miss the highest grade of disease [14], and there is evidence of considerable inter- and intraobserver disagreement between colposcopic assessments [15]. This results in great variation in management and increases the risk for both underdiagnosis and overtreatment.

Despite the importance of removing precancerous lesions with the potential for malignant transformation being beyond dispute, management decisions need to be balanced against the consequences of excising cervical tissue. To aid the process, individual circumstances such as general health (e.g., immunosuppression), lesion grade and size, age, parity, family planning, and the patient's wishes need to be considered. This personalised assessment and decision-making requires a fine balance of diagnostic procedures followed by adequate discussion and counselling of the patient. The use of technology, to not only improve but also standardize diagnostic abilities by adding objective measurements and improving documentation, may optimize this process.

The Dynamic Spectral Imaging (DSI) colposcope (DYSIS by DYSIS Medical Ltd, Edinburgh, UK) is a digital video colposcope that integrates the adjunctive DSI cervical mapping with standard colposcopy. DSI helps standardize the way colposcopy is performed (with respect to distance, illumination, field of view, and timing), quantifies the acetowhitening by measuring its intensity and persistence, and has been shown to increase sensitivity [16–18].

Presuming colposcopy is satisfactory and no obvious high-grade lesion is identified, a reassuring DSI map can enhance the colposcopic impression, thus assisting in reducing overtreatment. In addition, the standardization of practice as well as the introduction of an objective measure and documentation of the full acetowhitening process could facilitate follow-up of younger women with high-grade lesions, who opt to minimize risks for poor future obstetric outcomes, rather than treating them.

## 2. Case Presentation

A 27-year-old female was referred with cytology showing high-grade changes (moderate dyskaryosis). She was a nonsmoker with no significant past medical history, nulliparous, and currently under the treatment of a fertility centre for male factor infertility, awaiting intracytoplasmic sperm injection.

During the consultation, different management options were discussed. Pending colposcopic assessment, possibly the traditional approach would be “see and treat”, that is, a large loop excision of the transformation zone (LLETZ) of the cervix at the time of colposcopy, assuming colposcopy was considered to show high-grade changes. In view of her nulliparous status, age, and the wish to preserve fertility and reduce any potential risk factors for future pregnancies, the patient was alternatively offered a colposcopy with directed biopsies. Should the histology show high-grade cervical precancerous changes (CIN2+), an excision with a view to remove these changes could be performed at a second visit (select and treat).

The colposcopy was performed by an experienced colposcopist, consultant gynaecological oncologist, using the DSI colposcope.

**Figure 1:**
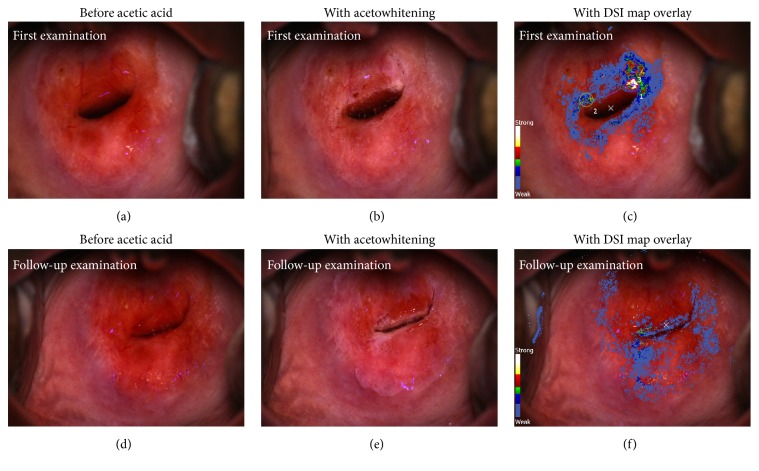
(a, b, and c) First examination. (a) Cervical appearance prior to application of acetic acid. (b) Cervical appearance after application of acetic acid. (c) DSI map overlaid on the cervix. Strong acetowhitening signal suggested potential high-grade lesion as identified by the DSI technology (colour legend on the left). The circular annotations are the biopsy sites selected and indicated during the examination; number 1 was identified by the colposcopist; numbers 2 and 3 were marked using the DSI map. (d, e, and f) Follow-up examination. (d) Cervical appearance prior to application of acetic acid. (e) Cervical colposcopic appearances after application of acetic acid. No significant acetowhitening effect is observed. (f) DSI map overlaid on the cervix, confirming the absence of significant changes. Notably, the area that yielded CIN2 at the first visit, now appears normal.

The colposcopic impression was of low-grade changes (Figures 1(a) and 1(b)), whilst the DSI mapping suggested that the acetowhitening changes potentially corresponded to high-grade CIN (Figure 1(c)).

In view of clinical and standard colposcopic appearances consistent with bacterial vaginosis and low-grade changes only, treatment was not offered on the day. Three directed punch biopsies were taken, a high vaginal swab was obtained, and a course of topical Clindamycin cream as empiric treatment of bacterial vaginosis was prescribed.

**Figure 2:**
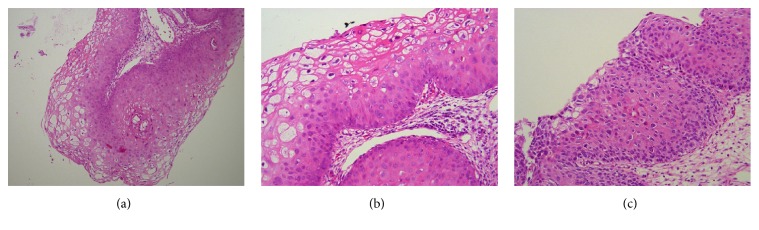
Histopathology images from the punch biopsies. (a) Mild atypia (CIN1) in lower third of cervix squamous epithelium with viral cytopathic effect in upper two-thirds (HE ×100). (b) Higher magnification (HE ×200) of CIN1 showing viral cytopathic effect in upper layers of squamous epithelium and mild atypia in lower third. (c) CIN2, showing almost full thickness epithelial atypia (HE ×200).

One of the biopsy sites was selected based on clinical judgement prior to reviewing the DSI map, and two further were directed by the DSI map (Figure 1(c)). The biopsy which was based on colposcopic impression showed koilocytic changes and CIN1 only, whereas the two biopsies based on the DSI evaluation showed CIN1 and CIN2 (Figure 2). There was no evidence of CIN3, cervical glandular intraepithelial neoplasia, or invasive malignancy.

Following these results, a further clinic appointment was arranged and the patient was reexamined with the DSI colposcope. The colposcopic images and DSI map from the first visit were reviewed prior to examination to provide a reference standard. This time, neither the colposcopic impression nor the DSI map suggested any areas suspicious of high-grade disease (Figures 1(d), 1(e), and 1(f)). Notably, none of the sites biopsied at the first visit demonstrated any features in keeping with high-grade lesion neither did the DSI map, suggesting that punch biopsies likely removed all high-grade disease initially present. Therefore, the patient was offered the option of conservative management by cytology and colposcopy at 6 months versus the standard LLETZ treatment and was fully counselled, in the presence of her partner, regarding available evidence and potential risks. Pending the infertility treatment, risks such as cervical stenosis and the possibility of requiring a test run prior to embryo implantation were stressed out in particular. However, in order not to delay commencement of fertility treatment, immediate cervical excision was opted for.

A LLETZ was performed with written consent, using the DSI colposcope, under local anaesthetic. Haemostasis was achieved by ball diathermy on the cervical crater as per standard practice. The specimen measured 20 × 18 × 11 mm and weighed 1.9 grams. Final histology showed HPV-related features and CIN1 clear from all margins (endocervical, ectocervical, and deep lateral). There was no evidence of high-grade CIN or invasive cancer.

The patient was discharged back to her family doctor, with a plan for cytology and high-risk HPV cotesting in six months as a test of cure, according to the National Health Service Cervical Screening Programme guidelines.

## 3. Discussion

Despite organised cervical screening programmes having proven their significant value in preventing cervical cancer, there is scope for further improvement in diagnostic accuracy. Excisional treatment for CIN2 and CIN3 is at present the gold standard and is of considerable value in prevention of transformation of these lesions into cancer. However, in view of the low colposcopic accuracy, there is a risk of overtreatment and increase in unnecessary morbidity. There is evidence that CIN2, if left untreated, regresses spontaneously in roughly half of the cases within two years [7–9]. Hence a more conservative approach might be suitable for some women, especially those with fertility wish.

Novel technology can support the clinical diagnosis and subsequent management. Several methods, such as Dynamic Spectral Imaging, optical spectroscopy, or optical coherence tomography, have been developed aiming to aid colposcopic assessment and evaluated in regard to their clinical and economic impact [19]. The DSI technology was shown to increase the sensitivity of colposcopy to 88% when used adjunctively to standard colposcopy [17], whilst also being cost-effective [19].

The case presented above demonstrates that the sensitivity of colposcopy when combined with DSI was indeed higher than colposcopy alone, both in terms of forming the right impression and identifying the sites to biopsy. The DSI-guided biopsy led to detection of high-grade CIN, whereas the colposcopic impression of low-grade would have otherwise been confirmed by the colposcopically directed biopsy which indeed showed CIN1.

The ability to review images and the DSI map from the first visit, performed under identical circumstances with those in the second, enabled the colposcopist to confidently advice the patient towards conservative management. The LLETZ was performed due to patient's wish and in accordance with standard guidelines following high-grade CIN on directed biopsies. The fact that the histological assessment of the treatment specimen showed no high-grade CIN illustrates that this case could have indeed been managed more conservatively, decreasing any risks associated with the excision.

This case highlights that the opportunity to compare images and DSI maps side by side, and potentially quantify cervical changes, which can be particularly valuable when patients are not seen by the same colposcopist over time, may prove invaluable in optimizing care for the selected group that will prefer conservative management to excision, and the development of appropriate guidance.

It also underlines the offered advantage to be more reassured when both colposcopic impression and the DSI map are negative for high-grade CIN, as the high negative predictive value with the use of DSI suggests [17, 18], and thus reduce unnecessary treatments.
